# Relationship between DLK1 gene promoter region DNA methylation and non-small cell lung cancer biological behavior

**DOI:** 10.3892/ol.2020.11577

**Published:** 2020-04-27

**Authors:** Zhaokui Zhong, Yongting Ye, Wei Guo, Yi He, Weicai Hu

Oncol Lett 13: 4123-4126, 2017; DOI: 10.3892/ol.2017.6019

An interested reader drew to our attention that data featured in Figs. 3 and 5 of the above paper appeared to have been previously published in the journal *PloS One* [Li L, Tan J, Zhang Y, Han H, Di X, Xiao T, Cheng S, Gao Y and Liu Y: DLK1 promotes lung cancer cell invasion through upregulation of MMP9 expression depending on Notch signaling. PLoS One 9: e91509, 2014] and in a Chinese doctoral dissertation.

Following an internal investigation, the Journal was able to confirm that this accusation of plagiarism was well-founded. On those grounds, the Editor of *Oncology Letters* has decided to retract this paper. Every effort was made to contact the authors in this regard, but without success. The Editor deeply regrets the inconvenience that this retraction has caused to the authors of the previously published articles, and to the readership of the Journal.

